# Cerebrospinal fluid production by the choroid plexus: a century of barrier research revisited

**DOI:** 10.1186/s12987-022-00323-1

**Published:** 2022-03-22

**Authors:** Nanna MacAulay, Richard F. Keep, Thomas Zeuthen

**Affiliations:** 1 Department of Neuroscience, University of Copenhagen, Blegdamsvej 3, 2200 Copenhagen, Denmark; 2 Department of Neurosurgery, University of Michigan, Ann Arbor, MI USA

**Keywords:** Cerebrospinal fluid, CSF, Choroid plexus, Blood-CSF-barrier, Osmotic gradients, Membrane transporters, Local osmotic forces, Transporter-mediated water transport

## Abstract

Cerebrospinal fluid (CSF) envelops the brain and fills the central ventricles. This fluid is continuously replenished by net fluid extraction from the vasculature by the secretory action of the choroid plexus epithelium residing in each of the four ventricles. We have known about these processes for more than a century, and yet the molecular mechanisms supporting this fluid secretion remain unresolved. The choroid plexus epithelium secretes its fluid in the absence of a trans-epithelial osmotic gradient, and, in addition, has an inherent ability to secrete CSF against an osmotic gradient. This paradoxical feature is shared with other ‘leaky’ epithelia. The assumptions underlying the classical *standing gradient* hypothesis await experimental support and appear to not suffice as an explanation of CSF secretion. Here, we suggest that the elusive local hyperosmotic compartment resides within the membrane transport proteins themselves. In this manner, the battery of plasma membrane transporters expressed in choroid plexus are proposed to sustain the choroidal CSF secretion independently of the prevailing bulk osmotic gradient.

## Introduction

Brain fluid homeostasis is critical in health and disease. Normal brain function is highly reliant on a spatial connectivity that may be altered with pathological brain fluid accumulation. Conditions such as stroke or traumatic brain injury may associate with elevated intracranial pressure, which, if left untreated, may lead to subsequent brain herniation and death. In addition, brain fluid imbalance occurs in hydrocephalus patients, in which a mismatch between cerebrospinal fluid (CSF) production and absorption may result in dilated CSF spaces with potentially devastating consequences. Although CSF secretion is a crucial component in brain fluid homeostasis, there is still uncertainty regarding the molecular mechanisms underlying its secretion. An understanding of those mechanisms is essential for development of novel therapeutic approaches. This review assesses evidence related to CSF secretion and posits that transporters expressed in the CSF-secreting tissue, the choroid plexus, play a central role in CSF secretion by their inherent ability to mediate transporter-mediated water transport.

## CSF and its flow through the ventricular system

**Fig. 1:**
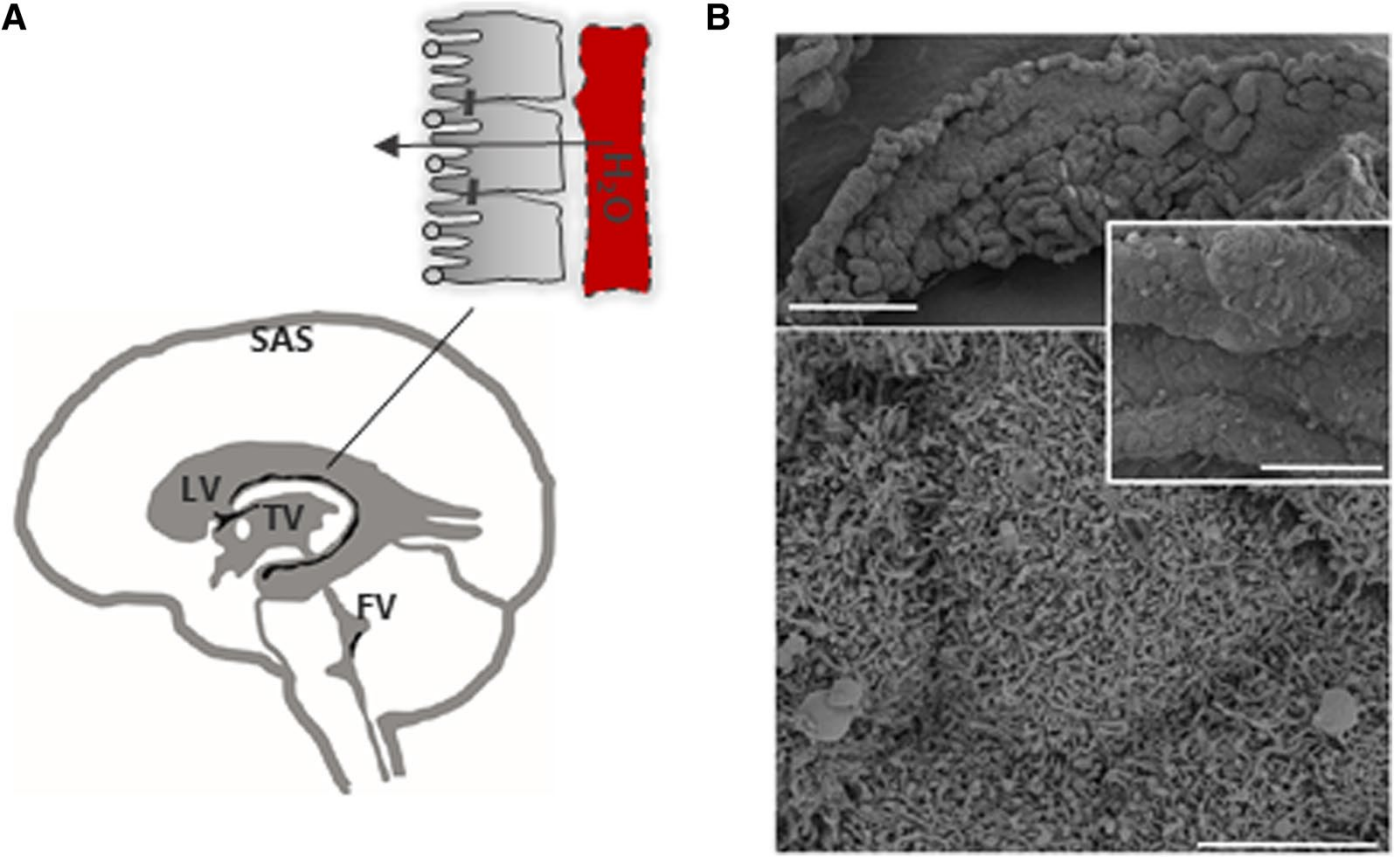
The CSF-containing ventricular system and choroid plexus. **A** The fluid-filled ventricular system and subarachnoid space (grey) with choroid plexus marked in black. LV; lateral ventricle, TV; third ventricle, FV; fourth ventricle, SAS; subarachnoid space. Inset illustrates the monolayer of choroid plexus epithelial cells bordering on the vasculature (in red) with tight junctions illustrated as bars and AQP1 as light grey spheres on the luminal-facing side. **B** Scanning electron microcopy images of rat choroid plexus illustrates the frond-like arrangement of the epithelium dictated by the ensheathment of blood vessels in the underlying connective tissue and the abundant and interdigitated microvilli. Scale bars 500 μm (top panel), 50 μm (insert), 5 μm (bottom panel). SEM images courtesy of Frederik Vilhardt, University of Copenhagen

The mammalian brain consists of approximately 80% water, which is the main constituent of the cerebrospinal fluid (CSF). The CSF is interconnected with the interstitial fluid (ISF) [1–4] that surrounds the individual brain cells and structures to provide a pathway for transport of nutrients, hormones, and metabolites [5]. The CSF envelops the brain to provide buoyancy and acts as an insulation from mechanical insult. In addition, the CSF fills the central ventricular system (Fig. 1A), which consists of the two lateral ventricles in the telencephalon that connect to the third diencephalic ventricle via the foramina of Monro. The aqueduct of Sylvius provides access to the fourth ventricle that is located between the dorsal face of the brainstem and cerebellum. The majority of the CSF travels from the fourth ventricle to the subarachnoid space via the foramina of Magendie or Luschka, while the remaining fraction enters the central canal of the spinal cord. The CSF is propelled through the ventricular system by a combination of hydrostatic pressure arising from its continuous production, by arterial pulsations, by the respiratory cycle, and by directional beating of cilia located on the ependymal lining of the ventricles and the aqueduct [6, 7]. In rodents, CSF reabsorption occurs mainly along the cranial nerve sheaths, predominantly those of the optic and olfactory nerves (the latter via the cribriform plate), to extracranial lymph nodes [8, 9]. Spinal nerve sheaths and the meningeal lymphatics contribute as additional CSF drainage sites [8–10]. The arachnoid granulations or villi protruding from the subarachnoid space into the dural venous sinuses were originally assigned as the predominant site of CSF drainage, but macroscopic arachnoid granulations are absent in lower animals and human fetuses [8, 11, 12]. Newborn sheep have similar absence of arachnoid granulations, but nevertheless displayed a comparable rate of CSF drainage to that of the adult animal [9]. An anatomical pathway of drainage through arachnoid villi is questionable [8]and the arachnoid granulations or villi thus may act as an additional drainage pathway that may be employed under conditions of elevated intracranial pressure [13].

## Anatomy of the ventricular system and the choroid plexus

The ventricular walls are lined by a layer of ciliated columnar ependymal cells, which serve to separate the ventricular cavities from the brain tissue [14]. Notably, the ventricular ependymal wall does not serve a secretory function [15, 16] and does not represent a diffusion-restrictive barrier in the adult. Thus, solutes can diffuse between brain parenchyma and the CSF contained in the ventricles, due to the lack of tight junctional coupling between ependymal cells [1–4]. At sites where the ependymal cell layer adheres closely to pia mater, tela choroidea arises [17]. This structure protrudes into each of the ventricular cavities as frond-like structures known as choroid plexus (Fig. 1B). The lateral choroid plexus originates from the floor of the central part of the lateral ventricle and continues into its temporal horn (choroid plexus is absent from the anterior and posterior horn of the lateral ventricle [17]). The tela choroidea located in the ceiling of the third and fourth ventricles give rise to the third and fourth choroid plexuses. The choroid plexus tissue is richly vascularized with the lateral choroid plexuses being supplied by the anterior and posterior choroidal arteries, which are branches of the internal carotid artery and the posterior cerebral artery, the latter of which also supplies the third choroid plexus. The fourth choroid plexus receives its vascular supply from the inferior cerebellar artery [6]. The choroidal capillary endothelial cells are fenestrated and surrounded by loose connective tissue of various thickness with interspersed stromal cells and fibroblasts [18, 19]. A continuous basement membrane separates the connective tissue from the monolayer of cuboidal choroidal epithelial cells, which are of approximately 10 µm height and width in the adult rat [14, 19], Fig. 1A. The epithelial cells are interconnected by tight junctions located at the lateral aspect of the cell membrane towards the luminal side of the epithelial cell layer [1, 20]. Various choroidal junctional proteins, including claudin -1, -2-,-3 -11, -19, occludin and ZO-1 [20–23], serve to prevent paracellular passage of larger molecules and thus permit the choroid plexus epithelium to act as a barrier between the blood and the CSF [1]. Choroid plexus mostly consists of epithelial cells [24, 25], but contains the endothelial cells of the capillaries in addition to various other cell types, such as fibroblasts and immune cells [25]. The choroidal surface may be adorned with choroidal macrophages, coined ‘epiplexus cells’ [14, 18, 26].

The choroid plexus epithelium displays general features of secretory epithelia with basolateral foldings and interdigitations, a luminal brush border consisting of short microvilli, a high density of mitochondria [18, 24, 27, 28], and a high transcript abundance of genes encoding membrane transport mechanisms [29]. The microvilli covering the surface of the choroid plexus are 1–3 µm long with a density of 7–18 microvilli per µm^2^, Fig. 1B [14, 24, 28, 30]. These microvillar protrusions increase the surface area around 15-fold across several animal species [19, 27, 30] and thereby enlarge the total choroidal surface area to approximately half of that of the brain capillary bed in the rat [19]. Taken together with the choroid plexus containing at least as many mitochondria as the brain endothelium [27], and a fourfold higher expression of the Na^+^/K^+^-ATPase α1 subunit [31], which is the dominant catalytic subunit in both barrier cell types [30, 32], the choroid plexus appears quantitatively geared for maintaining brain ion homeostasis and secreting CSF.

The lateral, 3rd, and 4th choroid plexuses appear qualitatively similar [18, 19, 24, 28], although the 4th choroid plexus of dog contains less connective tissue, which allows a closer connection between the vascular bed and the choroid plexus epithelial cells [18]. The 4th choroid plexus is the largest of the plexuses (38–47% of the total mass) in rat and dog, nearly double that of a single lateral choroid plexus (18–21% of the total mass), with a smaller 3rd choroid plexus (11–25% of the total mass) [33, 34]. Interestingly, the surface-to-volume ratio of the 4th choroid plexus of adult dogs is higher than the others (due to the lesser connective tissue), and the surface area of the 4th choroid plexus thus represents 55% of the total choroid plexus surface (38% for the two lateral choroid plexuses and only 7% for the 3rd choroid plexus) [18]. In addition, the 4th choroid plexus displays the most intense growth curve post-weaning [33], partly due to its robust increase in epithelial cell number [24]. Taken together with its 25% higher oxygen consumption per unit weight compared to that of the lateral choroid plexus [35], the 4th choroid plexus likely serves a key function in CSF secretion.

## CSF secretion by the choroid plexus

The choroid plexus was proposed as the origin of CSF secretion already in the mid-1800s [36, 37], and was experimentally demonstrated as such in the beginning of the 1900s with subsequent confirmation by complementary experimental approaches:

1. Blockage of the duct of Sylvius (the passageway between the 3rd and the 4th ventricles) or the foramina of Monroe (the passageway between the lateral and the 3rd ventricles) caused lateral ventricular enlargement in dogs [38, 39], which was limited to the one ventricle with a functional choroid plexus following a prior unilateral choroidal plexectomy [38].
2. Upon exposure of the lateral ventricle of anaesthetized and mechanically ventilated cats, filter paper placed on the surface of choroid plexus was rapidly saturated with fluid. In contrast, filter paper placed on the ependymal wall or the pia mater did not become appreciably moistened even after many minutes [16].
3. Choroidal fluid secretion was directly demonstrated in situ by successful collection of CSF from the surface of the exposed choroid plexus in the anaesthetized and mechanically ventilated cats [16]. This fluid was of similar composition to that of the bulk CSF collected from cisterna magna.
4. Fluid was lost from the vasculature through its passage through the choroid plexus of anesthetized rabbits to an extent matching the volume of secreted CSF [40].
5. Conventional inhibitors of CSF secretion (ouabain and acetazolamide, see later) efficiently reduced the CSF secretion when topically applied to the surface of the exposed choroid plexus in situ in anaesthetized cats [41] or rabbits (by measurements of fluid lost from the choroidal vasculature) [40].
6. Cultured or organoid choroid plexus epithelial cells secrete CSF in a self-contained compartment [42, 43].
7. Choroid plexus actively secretes Na^+^ [44, 45], which is coupled to fluid secretion in choroid plexus as well as in other epithelia [46–48]. Directional Na^+^ flux may therefore be considered a proxy of CSF secretion. Na^+^ rapidly enters the brain through the choroid plexus epithelium as evident by (i) swift (< 10 min) equilibration between ^24^Na^+^ introduced into the vasculature and the CSF collected directly from the surface of the exposed choroid plexus of cat [16], and (ii) swift appearance of vascular ^22^Na^+^ and ^36^Cl^−^ in the CSF [49], due to their higher effective permeability across the choroid plexus epithelium than across the capillary endothelium [50], but see [51].

It appears that around 30–40% of the cerebrospinal fluid is formed upstream from the aqueduct (by the lateral + 3rd choroid plexuses) in dogs and monkeys [34, 52, 53], which aligns to some extent with the 50–60% of the total choroidal mass assigned to the lateral + 3rd choroid plexuses [33, 34]. Although the mass of the 4th choroid plexus constitutes less than half of the total mass [33, 34], it represents 55% of the total choroidal surface area in dogs [18]. This larger, and more metabolically active [35], 4th choroid plexus appears to support approximately 70% of the total CSF secretion in dogs (measured in the ventricular compartment downstream of the aqueduct [34]).

## Extrachoroidal sources of CSF secretion

Despite the numerous lines of evidence in favor of CSF secretion predominantly originating from choroid plexus, with its signature features of a secretory epithelium, some researchers propose a lesser role for choroid plexus in CSF secretion (for review, see [51]). Such conclusions were generally reached based on indirect experiments following surgical dissection of select choroid plexuses. Most renowned of these are the studies by Milhorat and colleagues, who demonstrated a 30% reduction in CSF secretion and ^24^Na^+^ transfer from blood to brain in bilaterally lateral plexectomized rhesus monkeys [54, 55]. Notably, this reduction in CSF secretion compares to the ~ 40% of total choroidal mass that the excised lateral choroid plexuses constitute [33, 34]. The remaining CSF secretion thus may reflect CSF secretion by the 3rd and the large and metabolically active 4th choroid plexus [18, 35] that most likely continue their secretory action despite an inflatable balloon placed in the 4th ventricle during the experimental procedure [55]. As CSF recirculates in the brain, CSF secreted by the 3rd and the 4th choroid plexuses may well reach ventricular compartments following its passage through the subarachnoid space and interstitial pathways [4, 56] and contribute to the CSF secretion rates measured in the plexectomized monkeys. In addition, it remains unresolved whether the site of choroid plexus excision regenerates to form a tight barrier that prevents a remnant of lateral ventricular fluid secretion to contribute to the measured CSF secretion rates [55]. An earlier study on these laterally plexectomized rhesus monkeys concluded that the hydrocephalus formation was ‘only slightly less pronounced’ in these monkeys compared to their control counterparts [54], and therefore that ‘the choroid plexus is probably not the sole or even the major source of cerebrospinal fluid within the primate ventricular system’. However, despite the high number of experimental monkeys (150 Rhesus monkeys) utilized for this study, their ventriculomegaly was not quantified and only a few representative images  are displayed in the article [54].

The ependymal cell layer lining the ventricular walls has been proposed as an alternative source of CSF over the years [57, 58], although direct probing of the exposed ependymal cell layer in anaesthetized cats left the filter paper barely moistened, in contrast to that in contact with the choroid plexus in the same preparation (see above, and [16]). The adult ependyma lacks tight junctional coupling and therefore does not represent a barrier [1–4]. This cell layer, accordingly, cannot be a site of active fluid secretion. Therefore, fluid that may enter the ventricles by diffusion or bulk flow across this cell layer must originate from the interstitial fluid, either from recirculation of choroidally secreted CSF, arising as a product of brain cell metabolism [57, 59], or via endothelial secretion [60] or filtration [61].

The interconnection of the interstitial fluid and the CSF [4, 5] has prompted suggestions of active fluid secretion across the cerebral endothelium (the blood–brain–barrier), in addition to that arising from the choroid plexus epithelium [51, 52, 60, 61]. Rapid exchange of radioactively labelled water across the endothelium [62] has occasionally been interpreted to represent fluid transport across the BBB, for review, see [51]. However, these measurements do not represent net fluid secretion across the endothelium, but rather diffusional exchange of water with that of the unlabeled water molecules. Notably, the capillary endothelium has a very low osmotic water permeability [63, 64], due to the absence of aquaporins (AQPs) in the cerebral vasculature [65, 66] and a high endothelial electrical resistance representing poor transendothelial permeability towards ions [67, 68]. The cerebral endothelium, however, express ‘epithelia-like’ polarized distribution of various transport proteins, which could partake in fluid secretion, for reviews, see [69–72]. However, the slow penetration of ^22/24^Na^+^ across the endothelial layer into the adjacent parenchyma [50, 73], and the inability of general inhibitors of CSF secretion and of various Na^+^-coupled endothelial transport mechanisms to prevent Na^+^ and fluid entry into the brain [48, 74], suggest that the cerebral vasculature may provide but a minor contribution to CSF secretion under physiological circumstances.

## CSF secretion independently of conventional osmosis

**Fig. 2:**
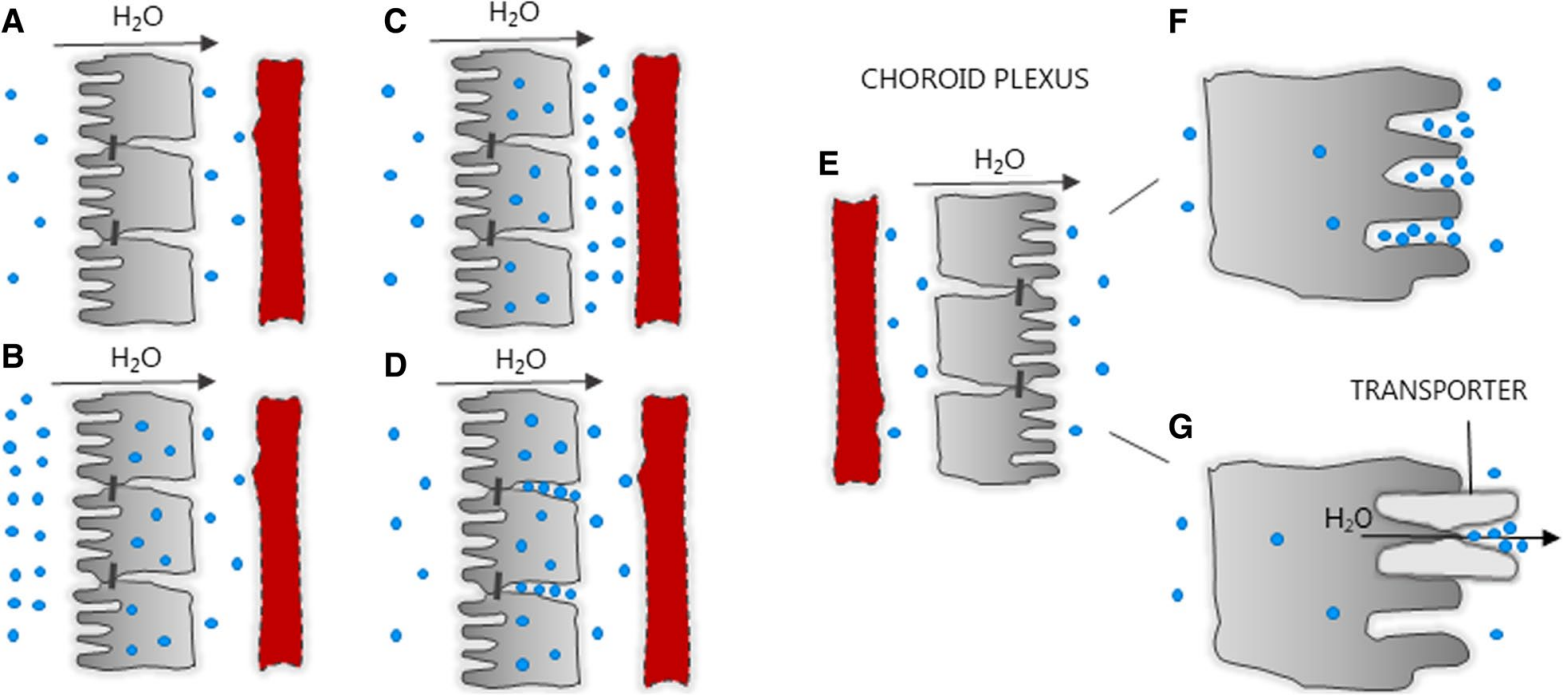
Models of isotonic secretion across leaky epithelia. **A** Epithelia can secrete isotonic fluid, i.e. can be bathed in solutions of identical osmolarity on both sides, and yet secrete fluid of a comparable osmolarity across the cell layer. **B** Epithelia can transport fluid in the *opposite direction* to that of an applied trans-epithelial osmotic gradient. **C** For net fluid transport to take place across an epithelium by a purely osmotic mechanism, it would require that the osmolarity of the cell interior surpasses that of the compartment from which the fluid originates, and that the osmolarity of the compartment into which the secretion takes place exceeds that of the cell interior. With few exceptions, however, such a stepwise osmolarity has not been found. **D** To circumvent the absent transepithelial osmotic gradient, it was suggested that the lateral spaces between the epithelial cells could act as a form of reservoir for electrolytes (osmolytes) secreted from the cell and prevented from leaving these spaces due to diffusion restriction. Such lateral intercellular space elevation in osmotic particles has not been detected (see below). **E** The choroid plexus is considered a ‘reverse’ epithelium, in a sense that the tight junctions are located on the luminal side, towards which the fluid is transported. The lateral intercellular spaces thus face the ‘wrong’ aspect of the epithelium and the possibility of a standing gradient (osmolyte accumulation in lateral intercellular spaces), driving the fluid secretion, is eliminated. **F** The space between the short microvilli was proposed as a site of local hyperosmolarity, but refuted following mathematical modelling. **G** The local hyperosmolar compartment is hypothesized to reside in an intramolecular space inside the membrane transport proteins themselves. For references, see text

**Fig. 3:**
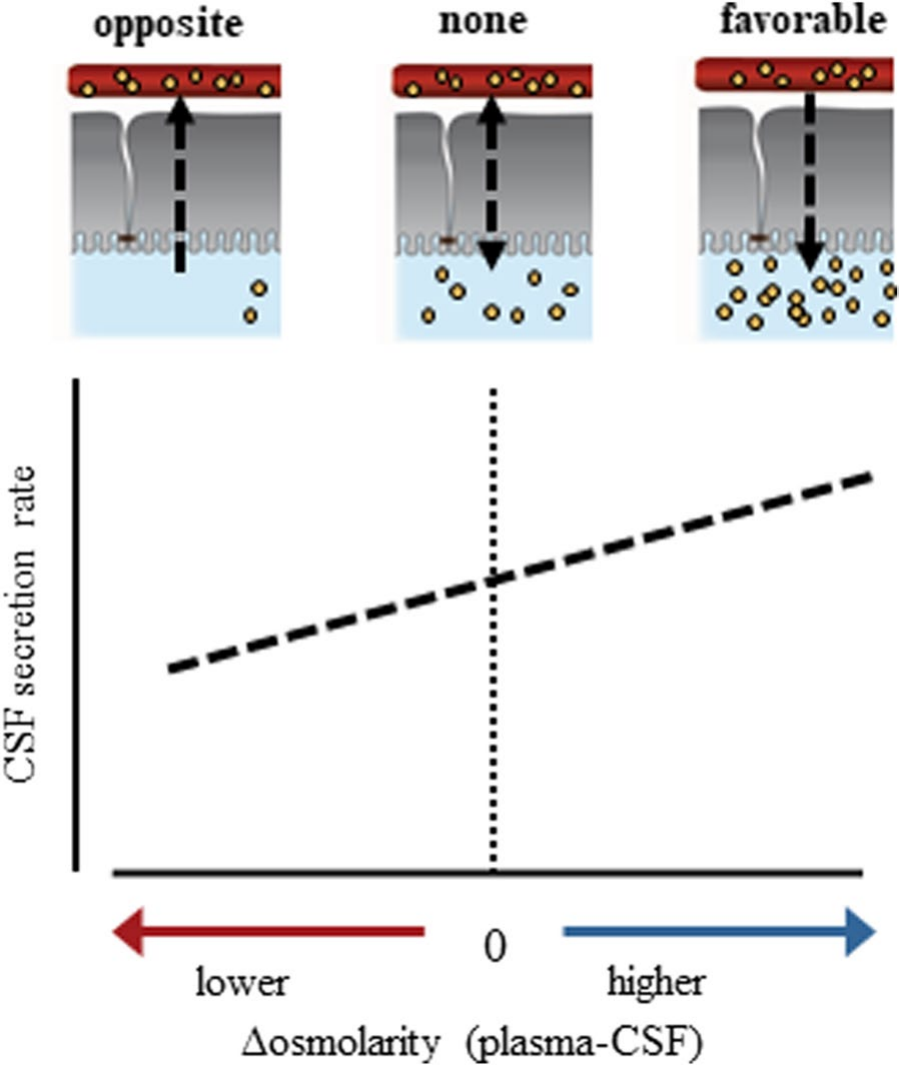
CSF secretion against an osmotic gradient. CSF secretion measured with different transchoroidal osmotic gradients demonstrate the ability of the choroid plexus to secrete CSF in the absence of an osmotic gradient (dotted vertical line) and even when faced with an osmotic gradient in the opposite direction to that of the CSF secretion (left side of the panel). The graph is adapted from [69] and based on published experimental findings in rabbit, cat, goat, and rat [30, 58, 90–93, 208, 209]

The CSF does not arise as a product of ultrafiltration from the plasma, but is generated by active fluid secretion [16, 75–77]. CSF secretion occurs at similar rates per unit choroidal weight in the tested mammalian species [77], a rate which readily compares to other highly secretory epithelia of the mammalian body [78]. The choroid plexus is a secretory epithelium of the’leaky’ kind, belonging to the class of epithelia encompassing the gall bladder, the small intestine, and the kidney proximal tubule [45, 79]. In the late 1800s, it was demonstrated that an epithelial cell layer could support fluid transport in the absence of an external driving force [80, 81], which was later confirmed in a range of different epithelia, i.e. toad skin, small intestine, gall bladder, and proximal tubule [82–85]. Epithelia, therefore, have the ability to secrete isotonic fluid, i.e. an epithelial cell layer can be bathed in solutions of identical osmolarity on both sides, and yet secrete fluid of a comparable osmolarity across the cell layer—over a wide range of bath solution osmolarities, Fig. 2A [79, 86, 87]. Later these findings were extended to demonstrate that these epithelia could transport fluid in the *opposite direction* to that of an applied trans-epithelial osmotic gradient, Fig. 2B [85, 88, 89]. Such ability to transport fluid independently of—and even against—an osmotic gradient is shared by the choroid plexus epithelium, across which there is no appreciable osmotic gradient [30], for review, see [69]: CSF secretion readily proceeds even when the choroid plexus epithelium faces large oppositely–directed osmotic gradients, which would favor fluid loss from the ventricle to the vasculature (Fig. 3), [30, 90–93]. ‘Uphill water transport’ thus appears to be a general feature of epithelia, but the underlying molecular mechanisms allowing such paradoxical trans-epithelial fluid transport independently of—or against—an osmotic gradient remain unresolved.

### Standing gradients in lateral intercellular spaces

The transport of electrolytes and other solutes across epithelia is energized directly by ATP-driven pumps and various other transport mechanisms, to which water transport is coupled. It has generally been assumed that fluid enters the epithelial cell by conventional osmotic driving forces and leaves the cell at the opposite membrane in the same manner. Such a transport model would require that the osmolarity of the cell interior surpasses that of the compartment from which the fluid originates, and that the osmolarity of the compartment into which the secretion takes place exceeds that of the cell interior (Fig. 2C). Epithelial cells are slightly hyperosmolar compared to their surrounding fluids [45, 94, 95], which would aid fluid entry into the cell. However, a permissive stepwise increase in osmolarity from the cell interior to the secreted fluid compartment (across the ‘exit’ membrane) is usually not apparent, thus challenging conventional osmotic water transport as the mode of fluid exit from the epithelial cell. To circumvent this challenge of fluid transport across the exit membrane, the central hyperosmolar compartment has been assigned to various other (micro)anatomical locations: the lateral intercellular spaces, the space between the microvilli, and finally an intramolecular space in the transport proteins themselves, which will be reviewed below.

The lateral spaces between the epithelial cells, or the underlying connective tissue [96], were suggested to act as a form of reservoir for electrolytes (osmolytes) secreted from the cell [97, 98], Fig. 2D. With an assumption that these spaces represented diffusive-restrictive compartments, exit of the electrolytes would be delayed. These electrolytes would thereby accumulate therein to generate a ‘standing gradient’ of elevated osmolyte content, which could promote osmotic fluid flow across the exit membrane of the given epithelium [97, 98]. Such elevated osmolarity in the lateral intercellular spaces and/or underlying connective tissue was hypothesized to suffice for promoting fluid exit from a cellular compartment towards the exit compartment of comparable (or even lower [45, 94, 95]) osmolarity [98, 99]. Despite the apparent beauty of such hypothesis, exclusively based on mathematical modeling, it was built on several assumptions that later were disputed experimentally as well as mathematically:

1. The Na^+^/K^+^-ATPase driving the osmolyte accumulation should be located predominantly on the lateral aspects of the basolateral cell membrane and concentrated towards the tight junctional end [87, 98], which it is not [86, 100].
2. The lateral intercellular spaces should be diffusion-restricted to retain the transported osmolytes [98]. However, the geometry and structural organization of the lateral spaces do not support this hypothesis [101, 102].
3. The lateral intercellular spaces should be hyperosmolar compared to the fluid in the exit compartment [97, 98]. Thorough probing with ion-sensitive microeletrodes into these spaces demonstrated that it was not the case [95, 103].
4. For the standing gradient hypothesis to prevail, the osmotic water permeability of the basolateral aspects of the epithelial cell membrane had to be exceedingly large [87, 98, 99, 104], in fact orders of magnitude larger than measured experimentally [86]. Diamond and colleagues then proposed that the experimental measurements of epithelial osmotic water permeability were grossly underestimated due to unstirred layers at the epithelium surface, possibly residing in the connective tissue [96]. Such unstirred layer should then prevent an experimentally inflicted osmotic challenge to reach fully the actual membrane surface and thus mask the real epithelial water permeability and lead experimenters to arrive at artificially low water permeabilities [104]. However, experiments on a leaky epithelium stripped of the supportive tissue representing (part of) the unstirred layer, provided similar water permeability values to those obtained with the intact epithelium [101, 105]. In addition, osmotic water permeabilities obtained from vesicular preparations of epithelial tissue (stripped of putative unstirred layers) were comparable to those obtained on intact epithelium [106, 107]. Taken together, unstirred layer effects appear to be negligible in various epithelia [101, 106, 108–111] and the experimentally obtained osmotic water permeabilities thus appear to stand.

Standing gradients and unstirred layers are challenging to measure experimentally, and arose as theoretical phenomena in order to explain movement of water that could not be understood with conventional osmotic water transport as the only item in the experimenter’s toolbox. Many of the assumptions employed in these original theoretical considerations [98, 99, 104] have thus been refuted [95, 101–103, 108, 110, 111]. Unstirred layers and standing gradients of the thickness and osmolarity originally proposed [96, 98, 104] simply are not of the magnitude required to explain the isotonic fluid secretion of ‘forward’ epithelia, such as gall bladder, small intestine, and proximal tubule, that transport fluid from the lumen to the basolateral compartment.

The molecular challenge of moving fluid independently – or even against – a transepithelial osmotic gradient is even more taxing in a ‘reverse’ epithelium like choroid plexus: the choroid plexus epithelium secretes the cerebrospinal fluid from the basolateral compartment and into the cerebral ventricles. Here, the tight junctions are located at the ventricular side of the epithelium [1, 112], which leaves the lateral spaces facing the blood side (Fig. 2E). With this organization, osmolytes will not accumulate in the lateral intercellular spaces (as these are on the wrong side of the epithelium), and the possibility of a standing gradient driving the fluid secretion is eliminated. In addition, the ventricular compartment into which the CSF is secreted is free of connective tissue, which could constitute an unstirred layer. Unstirred layers on the luminal surface of choroid plexus was, in addition, experimentally refuted by ion-sensitive microelectrode measurements of time of onset (i) of cell volume changes with osmolytes with different diffusion constants, (ii) of change in Cl^−^ concentration 0–40 µm distance from the cell membrane following introduction of test solutions with variant [Cl^−^] [79, 106], and (iii) by identical K^+^ concentrations detected as the K^+^-sensitive microelectrode approached the luminal membrane (prior to entry into the cell) [45].

As an alternative, the inter-microvillar space at the apical surface of the choroid plexus was proposed to represent a smaller space of a similar function to that of the lateral intercellular spaces in the ‘forward’ epithelia (Fig. 2F). As such, these spaces could serve as a diffusion-restricted area supporting a build-up of a standing gradient that could drive the CSF secretion independently of the ventricular osmolarity [75]. Mathematical modelling, however, has demonstrated that short microvilli (like those of the choroid plexus) cannot provide a space with diffusion-hindrance and are thus unable to support the required build-up of osmotic particles in the inter-microvillar space [30, 102]. CSF secretion therefore does not appear to rely on a local osmotic gradient in the inter-microvillar space at the apical surface of the choroid plexus [30].

### Tight junctions as a fluid pathway

With the limited experimental and mathematical support for the standing gradient hypothesis, and even less so for the ‘reverse’ choroid plexus epithelium, the junctional complexes between epithelial cells have been suggested to be a route for trans-epithelial transport of salt and water [99, 105, 113–116]. It should be noted that the standing gradient hypothesis relied on impermeable tight junctions and that these two hypothesized routes of trans-epithelial fluid flow therefore should be considered incompatible [86], or nearly so [117]. The tight-junctional solute and water flow has been proposed to take place by various molecular models, including the mechano-diffusion model, the electro-osmosis model, and the claudin model (for review, see [78]). The latter model hinges on select isoforms of the claudins, tight junctional proteins, being permeable to ions and water [118–121], which may contribute to fluid absorption by the proximal tubule [122]. Claudin 2 may be of specific interest with its selective localization to the choroid plexus epithelium within the brain [20, 21, 23] and its ability to form cation pores [118, 119]. Claudin 2, in addition, allows paracellular water flow upon a large (100 mOsm) experimentally inflicted osmotic challenge, when such is generated with the non-permeable mannitol as the osmolyte, but less so with excess NaCl constituting the osmotic challenge [120]. Although claudin 2 deficient mice (claudin 2^−/−^) display no overt phenotype [119, 123], the relative importance of claudin-2 in transchoroidal cation and water flux merits investigation. The possibility of tight junction-mediated fluid flow thus remains viable, but its potential quantitative contribution to fluid flow, if any, remains unresolved [78, 86, 87, 99, 109]. Most importantly, to support a significant fraction of the CSF secretion across the choroid plexus, the unit water permeability of each tight junction should be exceedingly large, given that the junctional area is much (> 1000 fold) smaller than that of the choroid plexus luminal membrane surface area [78, 96, 102, 108, 109]. With such a putatively enormous water permeability of each small junctional complex, their pores would be anticipated large enough to allow substantial solute flow in addition. Such parallel solute flux obliterates the osmotic driving forces required to sustain tight junction-mediated CSF secretion [102, 108, 109], as osmosis requires that two compartments of different osmolarity are separated by a semi-permeable membrane. Notably, the isotonic fluid secretion supported by choroid plexus and other leaky epithelia is characterized by similar composition of the fluids bathing both sides of the epithelium and hence surround the tight junctions [107]. As a result, minor driving forces would arise across the tight junctions and consequently only minute fluxes are anticipated to occur across the choroidal tight junctional complexes.

### Transporter-mediated water transport

The molecular mechanisms that support the isotonic fluid transport across epithelia, as well as those supporting fluid movement against substantial osmotic gradients, have thus remained a conundrum that has puzzled epithelial researchers for more than a century. However, an alternative arose with the suggestion of shifting the local hyperosmolar compartment from the lateral intercellular spaces (where they could not be detected, see above) to a hyperosmolar cavity inside the membrane transport proteins themselves, Fig. 2G [124]. This idea arose from the experimental demonstration that fluid closely followed the solute transport in various cotransporters. Importantly, this coupling took place independently of the direction and magnitude of an experimentally-inflicted osmotic challenge [79, 125–131]. The ‘uphill’ fluid transport is thus energized by the ‘downhill’ flux of a cotransported solute, so-called secondary active water transport or cotransporter-mediated water transport with the following characteristics:

1. A fixed number of water molecules following the transported solutes at each transport cycle leads to an immediate onset of cell swelling with inwardly-directed activation of the cotransporter and cell shrinkage with outwardly-directed activation of the cotransporter [126–129, 132].
2. Osmolyte accumulation by other means than regular cotransport (gramicidin-induced Na^+^ flux or GAT1-mediated Li^+^ leak currents) fails to induce the immediate onset of cell volume changes and gives rise only to delayed cell swelling by osmotically obliged water flux [128, 129, 133].
3. An ability to move fluid against an experimentally inflicted osmotic challenge [127–129, 132, 134].
4. The fixed number of transported water molecules per transport cycle does not vary with the prevailing osmotic gradient across the cell membrane and the ensuing osmotic water flux [71, 127, 129].

Cotransporter-mediated fluid movement has been demonstrated (i) in several different tissues or cell types (e.g. choroid plexus epithelium, retinal pigment epithelium, mammalian cell lines and *Xenopus laevis* oocytes heterologously expressing the proteins of interest, (ii) by several different experimental techniques (e.g. ion-sensitive microelectrodes, fluorescence imaging, and optical cell volume monitoring), and iii) in a variety of cotransporters, i.e. the Na^+^-coupled glucose transporter (SGLT), the glutamate cotransporter (EAAT1), the GABA transporter (GAT1), the monocarboxylate cotransporter (MCT1), the iodide cotransporter (NIS), the Na^+^/K^+^/2Cl^−^ cotransporter (NKCC1), and the K^+^/Cl^−^ cotransporter (KCC)[125–130, 132, 135, 136], for review, see [137].

**Fig. 4:**
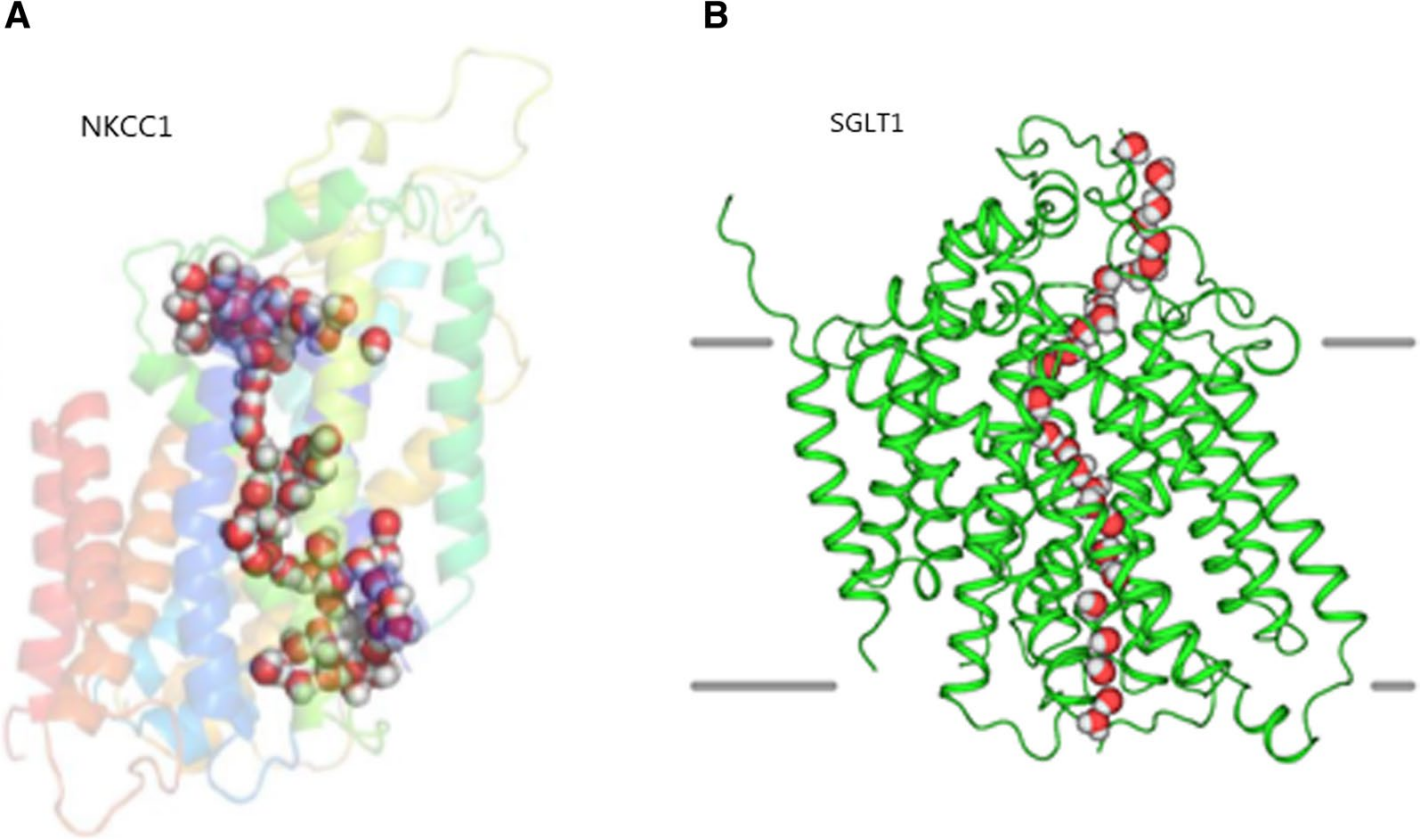
Water permeation pathways observed in cryo-EM structures of hNKCC1 and hSGLT1. **A** Sphere representation of selected water molecules in an apparent permeation pathway though the hNKCC1, figure from Supplementary data in [142]. The permeation pathway ends in a vestibule which contains about 40 water molecules. **B** Water permeation pathway in hSGLT1, illustrated by a representative simulation frame. Water molecules in red and white, figure from [143]

Despite the extensive experimental evidence demonstrating that cotransporter activity is closely associated with transmembrane fluid movement (see references above), the precise molecular mechanism of coupling remains elusive. The current suggested working model places the hyperosmolar coupling compartment [97] within the cavity of the protein itself (Fig. 2G). Upon release of the cotransported solutes from their central binding sites, they are in a thermodynamically free state during their passage through the exit cavity. In this manner, the exiting solutes are hypothesized to provide the compartmentalized hyperosmolarity required to promote fluid flow through the transport protein itself. Such a model has been proposed [106, 124] and is supported by mathematical modelling [138]. Molecular dynamics simulations have demonstrated net water transport through vSGLT in association with substrate release [139] or as stochastic fluctuations not strongly correlated with the motion of the exiting substrate [140]. Notably, a requirement for the hyperosmolar-cavity model to be viable is a continuous water-filled path through the protein, via which the water can permeate in response to the proposed hyperosmolar cavity. Studies in SGLT1 revealed that water shares the same pathway as the transported glucose molecule [141] and recent cryo-EM structures [142, 143] and molecular dynamics simulations [144] of the water-transporting cotransporters, SGLT1 and NKCC1, demonstrated such water-filled paths through their central parts and existence of water-filled entry and exit cavities, Fig. 4.

In the NKCC1 cryo-EM study, the authors concluded that their structure bore evidence *against* NKCC1-mediated water transport [142]. This objection, however, was based on an earlier working model that assumed cotransporter-mediated water transport occurring by occlusion of all the transported water molecules in an intra-proteinaceous cavity within the protein during the transport cycle (Dr. Forbush, personal communication). Although such a model was indeed speculated upon earlier [124, 145], it was abandoned > 10 years ago [106, 137]. The cryo-EM structures of NKCC1 and SGLT1 thus align well with the current hyperosmolar cavity working model for cotransporter-mediated water transport described above. Structural delineation of transporters residing in their different conformations are keenly awaited. These structures are anticipated to form the basis for molecular dynamics and mathematical modelling, which may allow identification of the intra-proteinaceous mechanisms supporting cotransporter-mediated fluid transport.

**Fig. 5:**
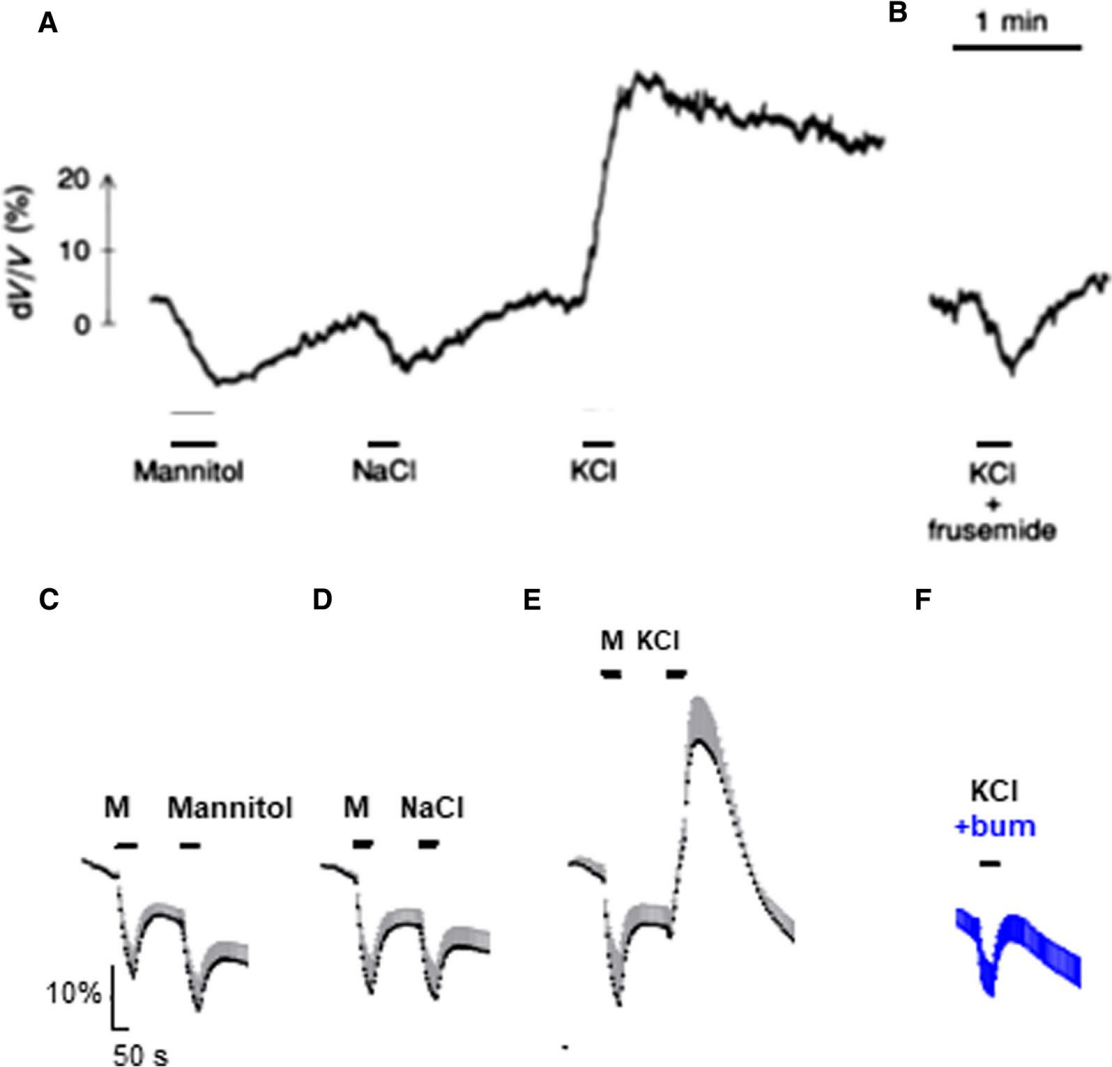
Transporter-mediated transport of water against an osmotic gradient. These experiments demonstrate that a sudden addition of KCl leads to an immediate inwardly-directed flux of water that proceeds against the osmotic gradient. In contrast, osmotic challenges by NaCl and mannitol, or KCl plus inhibitors of cotransporters elicit a simple osmotic shrinkage. **A** Cell volume changes recorded with ion-sensitive microelectrodes in salamander choroid plexus exposed to an osmotic challenge of 100 mOsm mannitol, NaCl, or KCl, as indicated in the figure. **B** Cell volume changes recorded with an osmotic challenge of 100 mOsm KCl with inclusion of furosemide (frusemide, 100 µM). Figure from [125] with permission. **C** Volume changes recorded in calcein-AM-loaded mouse choroid plexus challenged with a 100 mOsm gradient of mannitol (M), indicated by the bars. **D** Choroid plexus challenged with a 100 mOsm gradient of first mannitol (M), then NaCl. **E** Choroid plexus challenged with a 100 mOsm gradient of first mannitol (M), then KCl. **F** Choroid plexus challenged with 100 mOsm KCl in the presence of 10 µM bumetanide (n = 6 of each). Figure adapted from [146]

The ability of cotransporters to mediate water transport in a manner independent of an osmotic gradient has been demonstrated in ex vivo preparations of choroid plexus from salamander (*Necturus maculosus*) [79, 124] and from mouse and rat [30, 146] by experimentally promoting *inward* cotransporter-mediated transport with exposure to test solutions containing high [K^+^]_o_, Fig. 5. The acutely isolated choroid plexus shrunk when exposed to an osmotic challenge of 100 mOsm above that of the isotonic bath solution when the osmolyte was mannitol or NaCl. Identical experiments with KCl as the osmolyte promoted, instead, an instant and robust cell swelling due to the abrupt K^+^-induced onset of inwardly-directed cation-Cl^−^ cotransporter activity. Inhibition of the KCC (salamander) or the NKCC1 (rodent) abolished the K^+^-induced cell swelling and permitted the tissue to respond as a passive osmometer, as with mannitol and NaCl as the osmolyte, Fig. 5 [30, 125, 146]. These findings illustrate the ability of these transporters to move water in their transport direction, irrespective of the inflicted osmotic gradient. The ability of NKCC1 to transport water across the luminal membrane of the choroid plexus was reflected during its outwardly-directed transporter mode, by which it contributes to CSF secretion in mice, rats, and dogs (~ 50% of the total CSF secretion) [30, 146, 147]. The NKCC1-mediated CSF secretion rate increases with phosphorylation of the transporter by the Ste20-related proline-alanine-rich kinase (SPAK) [148, 149], which is amongst the highest expressed kinases in choroid plexus [150], where it co-localizes with NKCC1 [151]. NKCC1 appears to contribute to CSF secretion by its ability to promote transporter-mediated water transport and can, as such, be a missing link in the paradoxical ability of the choroidal CSF secretion to occur against an osmotic gradient. Other transport proteins in both the basolateral and the luminal choroid plexus membranes may well share this ability of moving water in the direction of their solute transport and thus contribute directly to the isotonic fluid secretion sustained by the choroid plexus and related epithelia.

## Choroid plexus transport mechanisms involved in CSF secretion

**Fig. 6:**
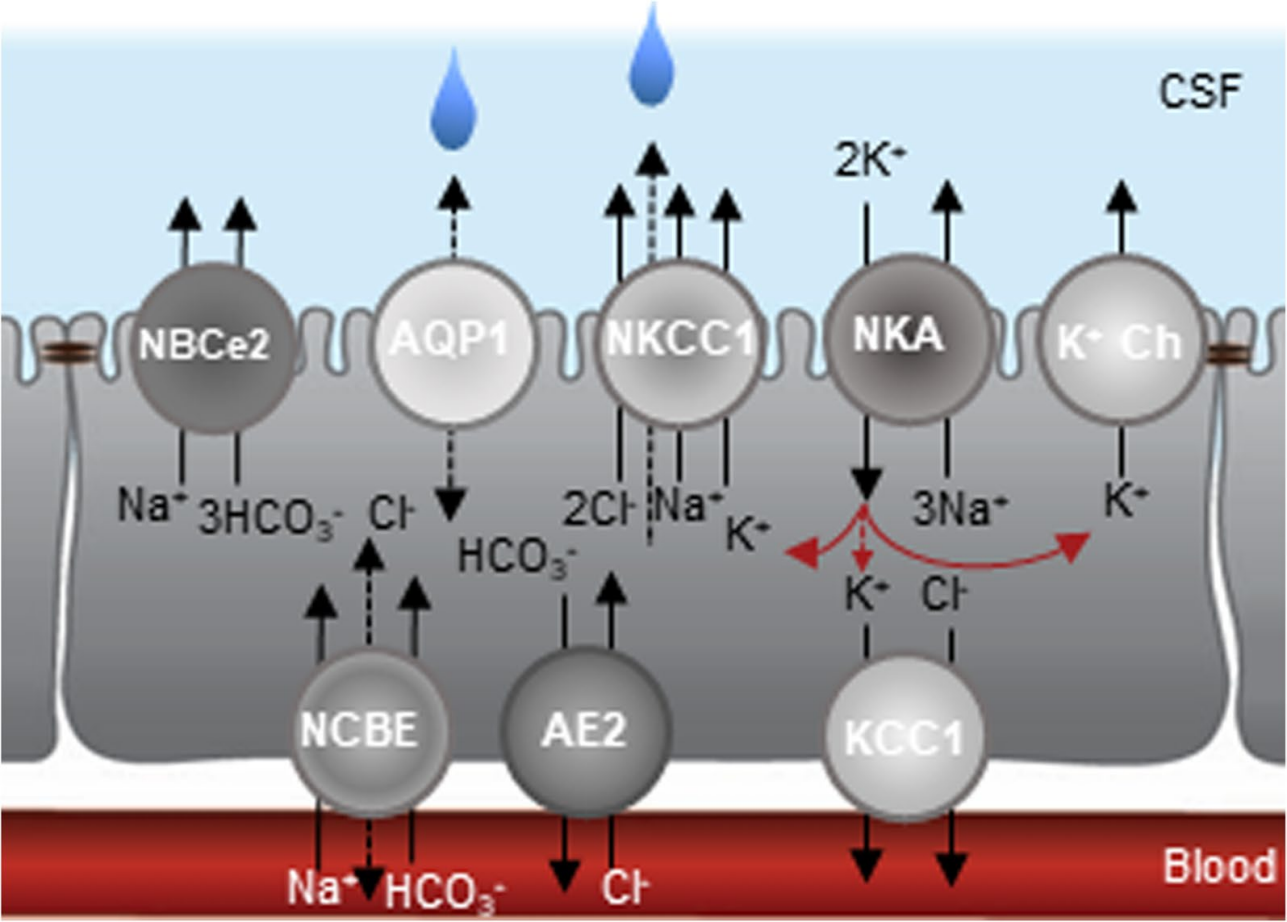
Choroidal transport mechanisms implicated in CSF secretion. Polarized localization of select transporters in the choroid plexus epithelium. NBCe2, NCBE, AE2; bicarbonate transporters, AQP1; aquaporin 1, NKCC1; Na^+^/K^+^/2Cl^−^ cotransporter 1, KCC; K^+^/Cl^−^ cotransporter 1, NKA; Na^+^/K^+^-ATPase, K^+^ Ch; K^+^ channels. Figure adapted from [146]. The read lines indicate a K^+^ shuttle with a potential efflux across the basolateral membrane via KCC1 in situations of high ventricular [K^+^]_o_ (dashed red line with small arrow head)

The choroid plexus is, like other epithelia, arranged with polarized expression of various membrane transport mechanisms. These transport proteins orchestrate the directional flux of ions and water that constitutes the CSF secretion (Fig. 6) and have been described in a number of earlier reviews. e.g. [51, 69, 152–154]. Although the quantitative contribution of the individual transport mechanisms remains unresolved, it is well established that Na^+^/K^+^-ATPase, NKCC1, and the HCO_3_^−^ transporters are key to CSF secretion [30, 69, 78]. The former two are both located on the luminal side of the membrane facing the CSF [146, 155–157]. Successful experimental inhibition of these transporters is accomplished solely by delivering their respective inhibitors directly into the ventricular compartment, which leads to reduced CSF secretion (ouabain (Na^+^/K^+^-ATPase inhibitor); [48, 158, 159], bumetanide (NKCC inhibitor)/furosemide (NKCC + KCC inhibitor); [93, 146–148]). Bumetanide thus fails to reach its target when administered intraperitoneally (i.p.) [160] and when administered intravenously (i.v.), bumetanide/furosemide only affects CSF secretion, if at all, indirectly by their diuretic action on the kidney and the ensuing diuresis[93, 148, 161, 162]. Notably, the inhibitory action of intracerebroventricular (i.c.v.) furosemide, which inhibits both NKCCs and KCCs, did not exceed the reduction in CSF secretion obtained with bumetanide [146], as was also observed for K^+^ efflux across the luminal choroidal membrane [30, 146, 163]. KCCs therefore do not contribute to ion and CSF flow across the luminal membrane of choroid plexus in accordance with the basolateral localization of KCC1 and negligible expression of KCC2-4 in choroid plexus [30, 146, 163–165].

The carbonic anhydrase inhibitor acetazolamide has long been known to reduce CSF secretion in experimental animals [41, 48, 166]. Acetazolamide is membrane permeable and thus appears to serve its action on intra- and extracellular carbonic anhydrases irrespectively of its mode of delivery (i.v., i.c.v., i.p.). Several isoforms of carbonic anhydrase are detected in the choroid plexus [167], and inhibitors thereof prevent the conversion of CO_2_ to H_2_CO_3_ thus limiting the formation of the HCO_3_^−^ required to drive the choroidal HCO_3_^−^ transporters. It should be noted, however, that this treatment affects all the widely expressed carbonic anhydrases and can cause severe systemic side effects in patients treated with this inhibitor to relieve elevated intracranial pressure [168]. It has therefore been speculated that the inhibitory action of acetazolamide may affect CSF secretion *indirectly* by altering choroidal blood flow, cell metabolism, blood pressure, kidney function, plasma [HCO_3_^−^] or pCO_2_, etc. [169–172]. However, recent experimental evidence illustrates the acetazolamide lowers ICP in rats by reducing CSF secretion via its inhibitory effect on choroidal carbonic anhydrases [167]. The choroid plexus expresses a battery of HCO_3_^−^ transporters, namely the electrogenic Na^+^-driven bicarbonate cotransporter NBCe2 in the luminal membrane, and the Na^+^-driven chloride bicarbonate exchanger NCBE and the anion (Cl^−^/HCO3^−^) exchanger AE2 as the dominant basolateral HCO_3_^−^ transporters (for review, see [153]), with a smaller contribution from the electroneutral Na^+^-driven bicarbonate cotransporter NBCn1 [78, 153, 164]. With the lack of specific inhibitors of the distinct HCO_3_^−^ transporters, their individual contribution to CSF secretion remains unresolved. The unspecific HCO_3_^−^ transport inhibitor diisothiocyanostilbene-2,2′-disulfonate (DIDS) demonstrated equally efficient inhibition (~ 30%) of CSF and Cl^−^ secretion irrespective of mode of delivery i.v. or i.c.v. [173], which supports the assignment of NCBE and AE2 as the basolateral Na^+^ and Cl^−^ loaders [50, 153, 174] and thus expected key contributors to fluid movement across the basolateral membrane. In support of the involvement of HCO_3_^−^ transporters in CSF secretion, mice with genetic deletion of NCBE or NBCe2 presented with reduced ventricular size in two of these animal models [175, 176], but not in another genetic mouse model deficient in NBCe2 expression [177]. However, the genetic modifications were associated with structural changes in choroid plexus, in addition to altered abundance or polarization of other choroidal transport mechanisms, i.e. AQP1, NKCC1, and the Na^+^/K^+^-ATPase [175, 178–180]. This secondary choroidal restructuring complicates the precise quantification of each of these choroid plexus transporters to CSF secretion (for review, see [153, 179]).

The water channel AQP1 is expressed at the luminal membrane of choroid plexus [155, 181]. It contributes substantially to the osmotic water permeability of that membrane, but CSF secretion is only reduced 20% in mice genetically deficient of AQP1 [182]. Humans genetically deficient in functional AQP1 display no neurological deficits [183, 184] and *AQP1*^−/−^ mice were not obviously different from their wild type counterparts, except for their reduced renal urinary concentrating mechanisms [185]. The systemic water homeostasis was thus altered in the *AQP1*^−/−^ mice, which taken together with a severe reduction in their central venous blood pressure [182] could indirectly contribute to the lowered CSF secretion and intracranial pressure. The direct quantitative contribution of AQP1 to CSF secretion thus remains unresolved. Future experimental efforts will reveal whether choroidal AQP1 aligns with other AQPs in a sense that their genetic deletion often bears little effect on the net fluid transport across various epithelia [186].

## K^+^ homeostasis by the choroid plexus

Apart from a role in CSF secretion, ion transporters in choroid plexus are also involved in regulating the ionic composition of the CSF. The excitable cells of the brain are highly sensitive to elevated K^+^ concentrations in the ISF/CSF ([K^+^]_ISF_/[K^+^]_CSF_), and the extracellular K^+^ concentration ([K^+^]_o_) is therefore tightly regulated [187, 188]. K^+^ is transported by some of the choroidal ion transporters implicated in CSF secretion, and these may contribute to the cerebral ion homeostasis, in addition to their role in brain fluid dynamics. The K^+^ concentration in the ventricles and the cerebral interstitial fluid is ~ 3 mM [187, 188]. This K^+^ concentration remains stable from the later part of gestation to adulthood, when measured by ion-sensitive microelectrodes in anesthetized rats (fetuses remained connected to the dams during the experimental procedure) of different maturities [3]. It should be noted that studies based on extraction of CSF postmortem arrive at higher values of [K^+^]_CSF_ (and plasma K^+^ concentration ([K^+^]_plasma_)) in neonates and fetuses [189–191], as [K^+^]_o_ rises quickly with anoxia in these age groups [192–194].

Adult mammals have a remarkable ability to sustain their ventricular and interstitial fluid K^+^ concentration, even with sustained plasma hyperkalemia [3, 187, 188, 195]. This control was absent in fetal rats [3], but arose in the forebrain parenchyma already at postnatal day 1 [3], likely due to endothelial Na^+^/K^+^-ATPase activity [32, 188, 195, 196]. In contrast, the ventricular [K^+^]_CSF_ management appeared gradually with development. Only after postnatal day 10, did the K^+^ concentration in the CSF remain stable with variable plasma [K^+^] in the rat [3], which aligns with the age-dependent increase in choroidal Na^+^/K^+^-ATPase abundance and activity [197, 198]. The rate of choroidal K^+^ clearance increased as a function of ventricular [K^+^]_CSF_ and was nearly abolished by the Na^+^/K^+^-ATPase inhibitor ouabain in vivo [199] and ex vivo [200]. The Na^+^/K^+^-ATPase is thus assigned as the dominant K^+^ uptake mechanism in choroid plexus [45, 163, 200]. In addition to an acute response to elevated K^+^ in the fluid surrounding the choroid plexus (in vivo/ex vivo), a chronic response to sustained hyperkalemia in rats promoted an elevation of choroidal expression of its Na^+^/K^+^-ATPase, of the associated ouabain-sensitive K^+^ uptake [201], and of the mitochondrial volume fraction required to sustain the elevated ATPase activity [202]. These responses appear to properly gear the choroid plexus to handle ventricular [K^+^]_CSF_ fluctuations.

Although the majority of the K^+^ influx into the choroid plexus epithelial cells across the luminal membrane is conducted by the Na^+^/K^+^-ATPase, a smaller ouabain-insensitive K^+^ influx was observed in rats [163, 200]. This fraction of K^+^ uptake was stable with age but increased with elevated [K^+^]_o_ [200]. Although the molecular identity of the mechanism supporting this smaller K^+^ uptake was not functionally assigned in the young rats [200], it could originate from inward transport by the NKCC1, which may reverse its transport direction with elevated [K^+^]_CSF_ [146, 163] or reduced choroidal [Na^+^]_i_ [203]. The NKCC1 abundance follows the developmental elevation of choroidal Na^+^/K^+^-ATPase expression [191, 197, 198]. Based on this increased expression, although with no direct functional evidence of NKCC1-mediated ion flux *into* the choroid plexus, NKCC1 was proposed to handle the choroidal K^+^ uptake underlying the [K^+^]_CSF_ management, and thus proposed as a novel CSF drainage pathway in early development [191].

In contrast, NKCC1-mediated efflux from choroid plexus across the luminal membrane to its surrounding fluid has been demonstrated ex vivo in choroid plexus of the adult mice, rats, and pigs by functional assays based on bumetanide-sensitive transport of K^+^ (^86^Rb^+^ flux) and Na^+^ (Na^+^-sensitive fluorescent dye) [30, 146, 163]. This NKCC1-mediated *outward* flux across the luminal cell membrane aligns with the contribution of NKCC1 to CSF secretion in dogs, mice, and rats [30, 146–148]. The ion content of the acutely excised adult mouse choroid plexus, compared to that of the surrounding CSF, predicts such outwardly-directed transport by NKCC1 [146]. Nevertheless, modulation of choroid plexus [Na^+^]_i_, potentially following enzymatic and mechanical isolation of choroid plexus epithelial cells followed by hours of culturing [203] or elevated [K^+^]_CSF_ in neonate mice [191], could well reverse the transport direction of NKCC1 under certain (patho)physiological conditions [204–207]. However, to render the choroid plexus a site of CSF drainage and thus permit net secretion of electrolytes and associated fluid from CSF to the vasculature [191], the NKCC1-mediated Na^+^ uptake into the choroid plexus epithelium across the luminal membrane must be paired with a Na^+^ efflux pathway across the basolateral membrane. With the Na^+^/K^+^-ATPase and the NKCC1 exclusively residing on the luminal membrane of choroid plexus, it remains unresolved how the Na^+^-coupled fluid exit is envisioned to occur across the basolateral membrane of choroid plexus [191].

The NKCC1-mediated K^+^ efflux from the choroid plexus epithelium into the CSF represents approximately two thirds of the K^+^ efflux from this tissue (in rat, pigs, and mice; [30, 146, 163]). The remaining K^+^ efflux across the luminal membrane occurs via K^+^ channels [45, 106, 163], the conductance of which is > tenfold higher in the luminal membrane than in that of the basolateral choroid plexus membrane [45, 106]. The rate of choroidal K^+^ uptake across the luminal membrane matches its rate of efflux across the same membrane [45]. K^+^ thus appears to be recycled across the luminal membrane in a sense that most of the Na^+^/K^+^-ATPase-mediated K^+^ uptake into choroid plexus leaves the cell across the same membrane [45], probably by the NKCC1 and the various K^+^ channels residing predominantly in the luminal membrane [45, 78, 146, 163], Fig. 6. In conditions of elevated ventricular [K^+^]_CSF_, a net efflux of K^+^ across the basolateral membrane may be required to sustain a stable [K^+^]_CSF_ [3, 199]. With the low K^+^ conductance in the basolateral membrane [45], one may envision a functional role of the outwardly-directed KCC1 that is detected on the basolateral membrane [146], Fig. 6.

## Conclusion

The past century of research on the ventricular system and the molecular mechanisms of CSF secretion peaked in the 1960s-1980s with a flurry of solid publications describing technically challenging in vivo experimentation on various animal models, performed by highly skilled and insightful physiologists. Although divergent viewpoints have emerged, direct experimental evidence supports that the choroid plexus is the dominant source of the CSF continuously secreted into the brain. CSF secretion occurs independently of conventional osmotic forces and readily continues in the face of an oppositely-directed osmotic gradients. This ability to transport water against an osmotic gradient does not arise from local osmotic forces, as those exemplified by the standing gradient hypothesis. This model lacks experimental support and is, in addition, poorly adaptable to the ‘reverse’ choroidal epithelium with its lateral intercellular spaces located on the ‘wrong’ epithelial aspect. Instead, emerging evidence suggests that it is membrane transport mechanisms with an inherent ability to couple fluid transport to their solute translocation that are the molecular motors driving CSF secretion. Future research aimed at identifying key transporters in the basolateral membrane of the choroid plexus and quantifying the contribution of the various choroidal transporters and their regulation in health and disease is envisioned to contribute to closing one part of the large knowledge gap that pertains to most aspects of brain fluid dynamics, i.e. CSF secretion, circulation, and drainage. Hopefully, such future knowledge will contribute new approaches towards regulation of brain fluid dynamics in pathological conditions such as hydrocephalus.

## Data Availability

Not applicable.

## Abbreviations

AQP: Aquaporin
CSF: Cerebrospinal fluid
i.v.: Intravenous
i.p.: Intraperitoneal
i.c.v.: Intracerebroventricular
DIDS: Diisothiocyanostilbene-2,2′-disulfonate
[K^+^]: K^+^ concentration
